# Hot Corrosion Behavior and Damage Mechanism on Yield Property of Nickel-Based Superalloy

**DOI:** 10.3390/ma18081749

**Published:** 2025-04-11

**Authors:** Xinyu Meng, Shaomin Lyu, Xingfei Xie, Chao Tang, Wugang Yu, Weixue Hou, Chengyu Wang, Jinglong Qu, Jinhui Du

**Affiliations:** 1 Research Institute of High-Temperature Materials, Central Iron and Steel Research Institute Co., Ltd., Beijing 100081, China; 2 Beijing GAONA Materials and Technology Co., Ltd., Beijing 100081, Chinasuperalloy_1@163.com (J.D.); 3 Sichuan CISRI-GAONA Forging Co., Ltd., Deyang 618000, China; 4 NCS Testing Technology Co., Ltd., Beijing 100081, China

**Keywords:** nickel-based superalloy, hot corrosion behavior, microstructure evolution, damage mechanism, tensile properties

## Abstract

Ni-based superalloys with enhanced environmental resistance at high temperatures are crucial for advanced gas turbine engines. The new polycrystalline nickel-based superalloy has excellent mechanical properties, but as a low-Cr, high-alloying superalloy, its environmental resistance has never been investigated. The hot corrosion behavior of the nickel-based superalloy under molten salt conditions and its effect on its tensile properties were investigated in this paper. The results showed the following: The diffusion of the Cr, Al, and Ni elements governs the majority of the corrosion process, resulting in the production of an environmentally damaged organization with internal sulfidation and surface oxidation. The Wagner model predicts the inability to form a dense Al oxide scale on the surface because the crucial generation condition of external Al oxides is not met. In addition, the growth stress in the damage scales is the main cause of cracking and spalling in the isothermal corrosion process. Due to the increased local stress concentration brought on by this environmental degradation, the sulfide scale acts as a fracture source, guiding the matrix cracking and influencing the tensile properties of the alloy.

## 1. Introduction

A superalloy refers to a material able to withstand large complex stresses at high temperatures above 600 °C and that has the surface stability of high-alloyed iron-based, nickel-based, or cobalt-based austenitic metal materials. Nickel-based superalloys refer to materials with high-alloyed nickel-based austenite as a substrate or nickel as the main element, as well as nickel–aluminum intermetallic compounds to produce high-temperature materials. Because of their superior fatigue and creep resistance, excellent high-temperature ductility, and strong resistance to oxidation and corrosion, nickel-based superalloys have emerged as the preferred material for critical aerospace engine components like turbine disks [1]. They occupy about 30–40% of the global aerospace engine material market. In addition, their market share in the non-aerospace sector is about 20–30%. Nickel-based superalloys contribute significantly to economic growth, and market demand is expected to continue to rise at a compound annual growth rate of 3–5%, with promising prospects for future development. Dynamic advancements in aero-engine technology have led to a heightened demand for an improved engine thrust-to-weight ratio, thermal efficiency, and service life. As a result, higher standards for the service temperature, high-temperature mechanical characteristics, and stability of superalloys have been established for turbine disks [2,3]. Advanced nickel-based superalloys are used in environments with temperatures higher than 700 °C. Since these alloys are used as hot-section components in aircraft applications that are exposed to fuel oil, oxidation, and marine atmospheric air streams, it will be interesting to see how they respond to environmental damage in the future. At this stage, by precisely controlling the nanomechanical properties of the material, the microstructure can be indirectly regulated, and the closure of micropores or the expansion of cracks can be observed by selecting different working conditions [4]. By optimizing the microstructure of the coating and reducing the formation of micropores and cracks, the hardness and abrasion resistance of the nickel-based gradient coating can be significantly improved, which provides a basis for the optimal design of the material [5]. Despite the fact that thermal barrier coatings, which are currently the main defense against high temperatures, can greatly extend service life [6], there are problems like coating–substrate thermal expansion mismatch, which can cause the coating or oxide scale to crack and spall, leading to failures [7,8]. Consequently, in order to quantify and regulate their mechanical properties during the service process, a thorough investigation of the environmental damage mechanism of the nickel-based superalloy material and a clarification of its impact on these mechanical properties is necessary. Following this, the environmental damage mechanism of the alloy must be predicted.

The deep and protective oxide scales of chromium (III) oxide (Cr₂O₃) and aluminum oxide (Al₂O₃) are primarily responsible for the exceptional resistance to environmental degradation exhibited by nickel-based superalloys [9]. Furthermore, the elements of chromium (Cr) and aluminum (Al) can slow down the rate of oxidation and hot corrosion [10,11]. Typically, nickel-based superalloys exhibit a very complex element diffusion behavior upon applied loads, which is caused by the environmental damage that occurs at high temperatures [10]. Although nickel-based superalloys can form protective oxides to enhance their corrosion resistance, the depletion of surface/near-surface alloying elements such as Cr, Al, and titanium (Ti) leads to the degradation of their properties, crack initiation, and fatigue failure [12,13]. The combination of temperature and stress can also lead the protective scale to break or be destroyed and can further oxidize and corrode the material [14,15,16]. Because of the higher service temperature of wrought superalloys, precipitation-strengthening elements are elevated, but solution-strengthening elements such as Cr are relatively low. Little research has investigated the environmental damage of low-Cr-content superalloys under service conditions at the maximum temperature of turbine disks. The structural changes in these alloys during the steady-state hot corrosion process, as well as the effect of the changes on the mechanical properties of the alloy, have never been investigated.

In order to study the hot corrosion mechanism of superalloys, researchers from both domestic and foreign universities currently use a certain mass fraction ratio of mixed salt Na_2_SO_4_ + NaCl for hot corrosion in crucibles and other containers covering the alloy surface. Mahobia et al. [17] found that the accompanying sulfur (S) element causes the sulfidation of Inconel 718 and GH4169 acicular δ-phase (Ni_3_Nb) and enrichment within the corrosion scale. According to research by Wei et al. [18], S can infiltrate the Cr_2_O_3_ oxide scale created by Inconel 600, diffuse into the matrix along grain boundaries, and mix with Cr to generate sulfide, which weakens the intergranular bond strength. However, S and nickel (Ni) in the alloy matrix combine to generate the low-melting-point compound nickel (II) sulfide (Ni_3_S_2_), which continuously melts when used and eventually causes sulfidation corrosion to develop. Birbilis et al. [19] used the XPS method to show that Rene104 has a rising amount of sulfur-containing compounds because there is not enough chromium oxide on the surface of the alloy during hot corrosion and that sulfur-related corrosion determines the damage. According to research by Nazmy [20] and others, the impact of sulfidation on the crack initiation process is responsible for the decrease in the LCF life of the IN738 alloy when exposed to synthetic ash and air containing sulfur dioxide/sulfur trioxide (SO_2_/SO_3_) at 850 °C-1000 h. Additionally, the fatigue behavior is impacted by anti-pitting and internal sulfidation that occur at the front of corrosion pits due to the erosive form of hot corrosion [21]. However, current research on superalloys under hot corrosion conditions mainly focuses on isothermal hot corrosion behavior, with less research on oxidation behavior and elemental competition mechanisms during the corrosion process. The effect of the evolution of the damaged organization on the mechanical properties during isothermal hot corrosion remains to be studied. An experimental alloy, as a high-alloying superalloy, will have a damaged structure and element diffusion mechanism that will be more complicated during the environmental damage process. Therefore, further study of the environmental damage mechanism and its effect on properties is necessary to promote the optimization of the alloy composition.

It is the first work to investigate the isothermal hot corrosion test of the alloy at 700–800 °C in molten salt of 75% Sodium sulfate (Na_2_SO_4_) + 25% Sodium chloride (NaCl) (wt % ratio). The hot corrosion behavior can be clarified by examining the corrosion kinetics, phase composition, and changes in the corrosion products of the alloy.

The influence of surface environmental damage on the tensile properties during the early stages of corrosion was studied. The purpose was to provide more guidance for understanding the corrosion–oxidation process of superalloys and evaluate strength degradation during service.

## 2. Materials and Methods

The experimental alloy was subjected to a triple process of vacuum induction melting (VIM), electro-slag remelting (ESR), and vacuum arc remelting (VAR). Then, the ingot was prepared for the homogenizing and forging process. The forging organization of 1130 °C solid solution treatment uses 850 °C and 760 °C double-stage aging to obtain the final heat treatment state specimen organization. The main chemical composition of the alloy is shown in Table 1 by ICP-AES.

The specimens (20 × 10 × 2 mm) were ground and polished according to HB5258-2000 [22]. Calcine the crucible until the change in mass is less than 0.0002 g. A 75% Na_2_SO_4_ + 25% NaCl (wt % ratio) salt mixture was placed in a crucible, keeping the molten salt just above the surface of the specimen for the hot corrosion test. Hot corrosion experiments were carried out in a muffle furnace in the air at 700, 750, and 800 °C for different times of 5, 10, 20, and 50 h. Under high-temperature salt bath conditions, the damage mechanism of new nickel-based superalloys in extremely harsh service environments was explored to initially explore the hot corrosion behavior and provide essential data for the next step of the long-term hot corrosion experiments.

The kinetic behavior in hot corrosion can be represented by the corrosion kinetic equation:

(1) $$\left(∆W\right)n=kp\cdot t$$

The weight change per unit area at the experimental temperature, the rate exponent, the corrosion rate constant, and the experimental time are represented by Δ*W*, *n*, *kp*, and *t*, respectively. After logarithmic treatment of both ends of the equation, the correlation corrosion rate constant can be found as follows:

(2) LnΔW=1/nlnKp+1/nlnt

After being immediately removed from the molten salts, the specimens were ultrasonically cleaned and rinsed with hot water to remove any remaining surface salt adhesions. Their mass changes were measured using an electronic balance that could weigh each specimen to within 0.0001 g. Three parallel specimens were put up for each hot corrosion condition to reduce experimental mistakes. Following the weighing process, the phase compositions of the corroded specimens were examined using an X-ray diffractometer operating at a scanning speed of 4° min^−1^ throughout the 10–100° range. Using Image-Pro Plus 6.0, a statistical analysis of the corrosion-related damage thickness of the scale was conducted. The Oxide Generation Gibbs Free Energy Change was calculated by HSC Sim 6.0 software. The thermophysical parameters required for simulation were calculated by JmatPro 7.0. The hardness of the sulfide scale was measured by nanoindentation device agilentG200 from KLA made in Milpitas, CA, USA. Scanning electron microscopy (JSM-7200F from JEOL Ltd. made in Tokyo, Japan) was used to characterize the surface and cross-section morphology of the hot corroded specimens. Energy-dispersive X-ray spectroscopy (EDS in JSM7200F) and an Electron Probe X-ray Micro-Analyzer (JXA-8530A from JEOL Ltd. made in Tokyo, Japan) were used to determine the elemental distribution of the oxidized specimens. Comparison of hardness of the environmental scale and substrate was obtained by the nanoindentation technique. GB/T228.1-2021 [23] requirements were utilized for the room-temperature tensile testing and mechanical characteristics of the corrosion specimen, with a dimension of M12 × φ5.

## 3. Results

### 3.1. Microstructure

**Figure 1:**
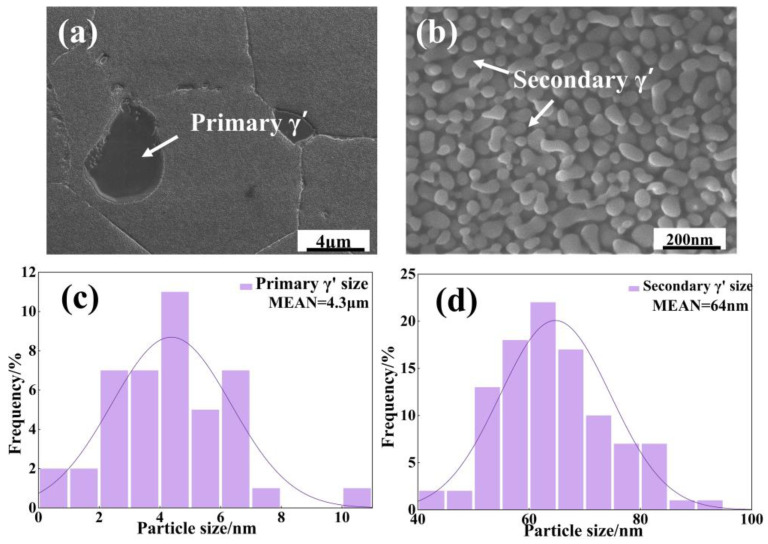
Characterization of the γ′ precipitation phase in the initial microstructure. (**a**,**b**) Microstructures; (**c**,**d**) average particle size of γ′ precipitation phase.

The initial γ grains and γ′ precipitation phase microstructure are presented in Figure 1, and GB/T6394-2017 [24] estimates that the average grain size of the γ grains is approximately 24.65 μm. The nanometer-sized, almost spherical secondary γ′ phase particles coexist with the micrometer-sized irregular primary γ′ phase particles.

In addition, the primary γ′ phases of the micrometer scale can be seen at both grain boundaries and grains. The near-spherical secondary γ′ phases of the nanometer scale can be observed in the vicinity of the primary γ′ phases, which are statistically derived to be 4.3 μm and 64 nm in average size (the standard deviation is 1.97 and 9.93) for each level of precipitated phases, respectively.

### 3.2. Corrosion Kinetics

**Figure 2:**
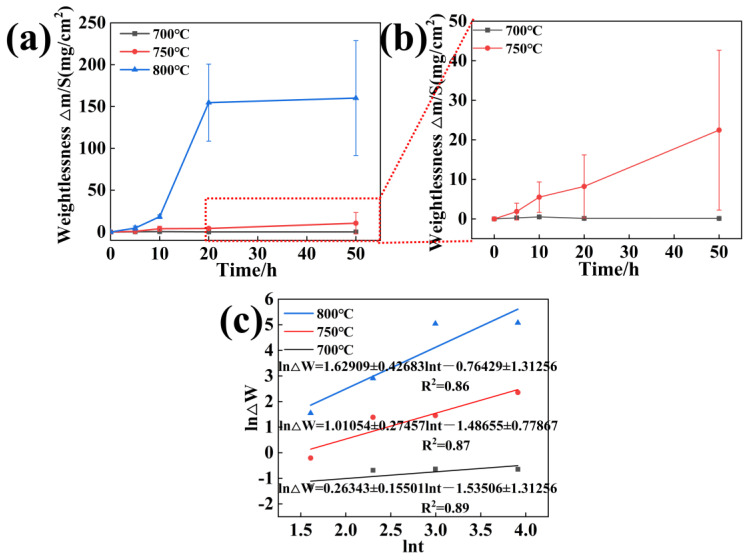
Relationship between mass variation and corrosion–oxidation parameters (**a**,**b**). Correlation between ln ΔW and lnt during hot corrosion. (**c**) Corrosion kinetic curves.

The relationship between mass variation and parameters is presented in Figure 2. The weight change of the experimental alloy is approximately 0.12441, 10.53473, and 160.10502 mg·cm^−2^ after 50 h of hot corrosion at 700, 750, and 800 °C. As 800 °C is close to the limit of the turbine disk alloy for the long-term service temperature, in the long-term salt bath conditions of the alloy, there is an inevitable surface thick corrosion layer shedding phenomenon. With the increase in corrosion time, the same experimental conditions of the weight of the individual workpieces under the weight change are relatively large. Figure 2b illustrates the link between the corrosion–oxidation parameters and mass change. The mass change rate of the experimental alloys increases with the increasing temperature.

As seen in Figure 2a, the corrosion rate exponents at the experimental temperatures—800 °C: 0.625536, 750 °C: 0.229685, and 700 °C: 0.002946—are derived by calculating the slope and intercept of the straight line by the data points in the fitted graph. As shown in Figure 2b, the alloy corrodes much more quickly at 800 °C than it does with molten salt at 700 and 750 °C. When combined with the corrosion weightlessness kinetic curve, the corrosion kinetic curve exhibits a linear law in the early stages of corrosion. In the late stages, however, the corrosion is greatly increased due to oxidation, which causes the corrosion kinetic curve to change to a parabolic mode. This is not due to the formation of a protective oxide scale on the alloy surface but is instead due to corrosion leading to the partial cracking and spalling of the damaged scales, and these things enhance the corrosion rate and promote the failure of the superalloy [25,26].

### 3.3. Phase Composition of Corrosion–Oxidation Products During Hot Corrosion

**Figure 3:**
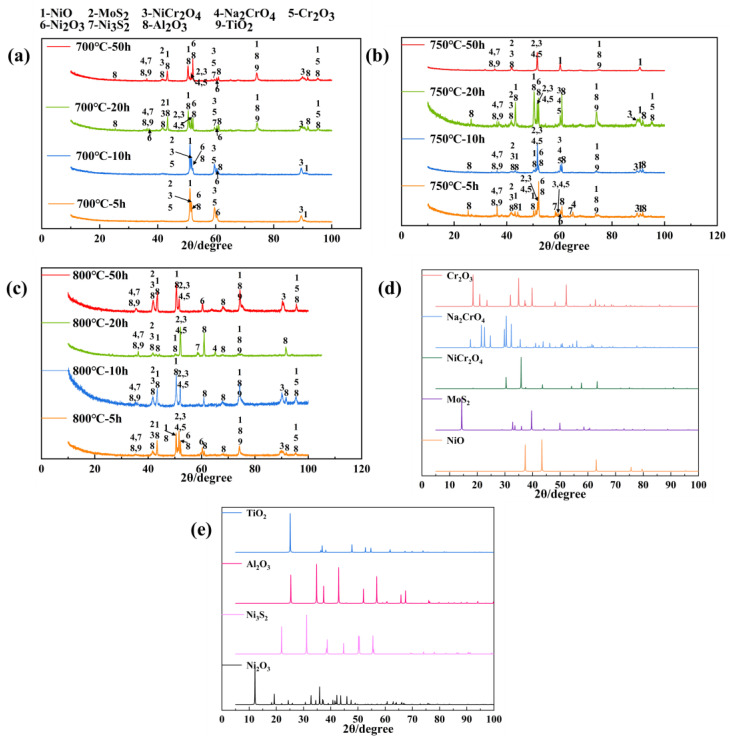
XRD pattern of hot corroded specimen at 700–800 °C. (**a**) 700 °C, (**b**) 750 °C, (**c**) 800 °C, and (**d**) and (**e**) represent theoretically simulated patterns based on the deposited crystallographic information files (cifs).

Figure 3 shows the phases that constitute the damage scales after corrosion. The environmental damage mode of the alloy is dominated by oxidation and sulfides under the hot corrosion condition of a molten salt environment at 700–800 °C. The principal products of corrosion are oxides of Cr, Ni, Ti, and Al, as well as sulfides of Ni and Molybdenum (Mo). The structure of the hot corrosion process is comparatively complex, starting with a reaction based on Cr, Ni, Al, and a small amount of the spinel structure of the oxide and Mo sulfide at 700 °C. Until the late stage of the process started showing signs of Ti oxides and Ni sulfides, the spinel structures (nickel (II) chromate) of the peaks gradually increased. Under the conditions of 750 and 800 °C, with the increase in the experimental time, the oxidation and sulfide product change trend was roughly similar to 700 °C; it is worth noting that due to the rise in temperature, the surface protective layer in the early stage of the experiment appeared with Ni_3_S_2_. This demonstrates that hot corrosion is a synergistic process of oxidation and sulfidation [27]. In particular, the type of corrosion products analyzed by the XRD physical phase analysis of corrosion products under hot corrosion conditions is less at the late stage of corrosion, and the corrosion weight change curve is in line with this outcome.

### 3.4. Corrosion Cross-Section Morphologies

**Figure 4:**
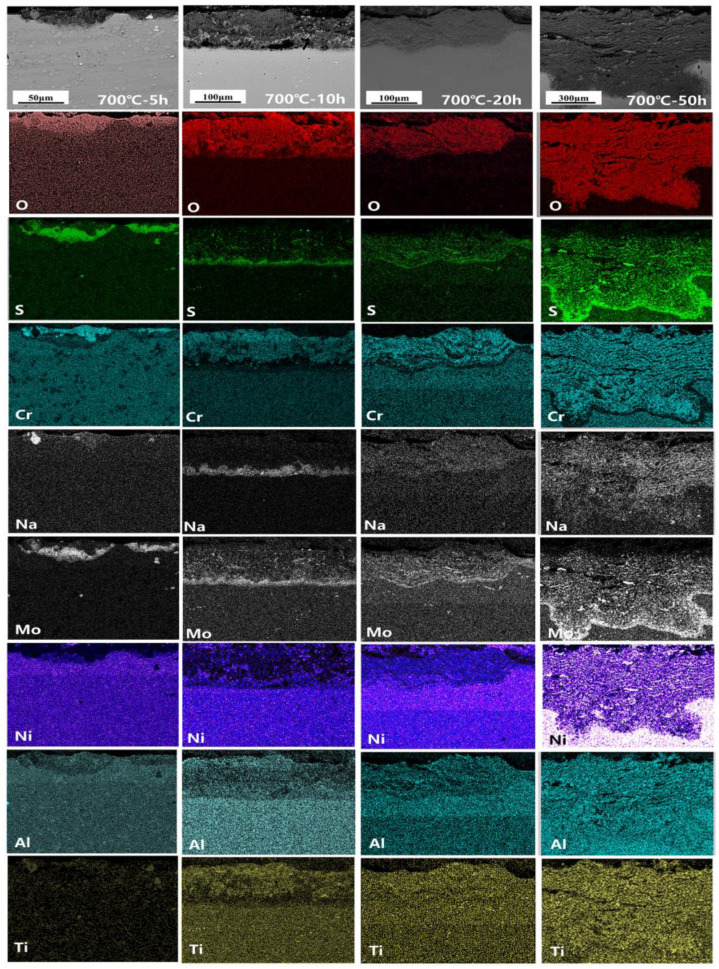
Cross-sectional morphology and elemental distribution of alloy at 700 °C.

**Figure 5:**
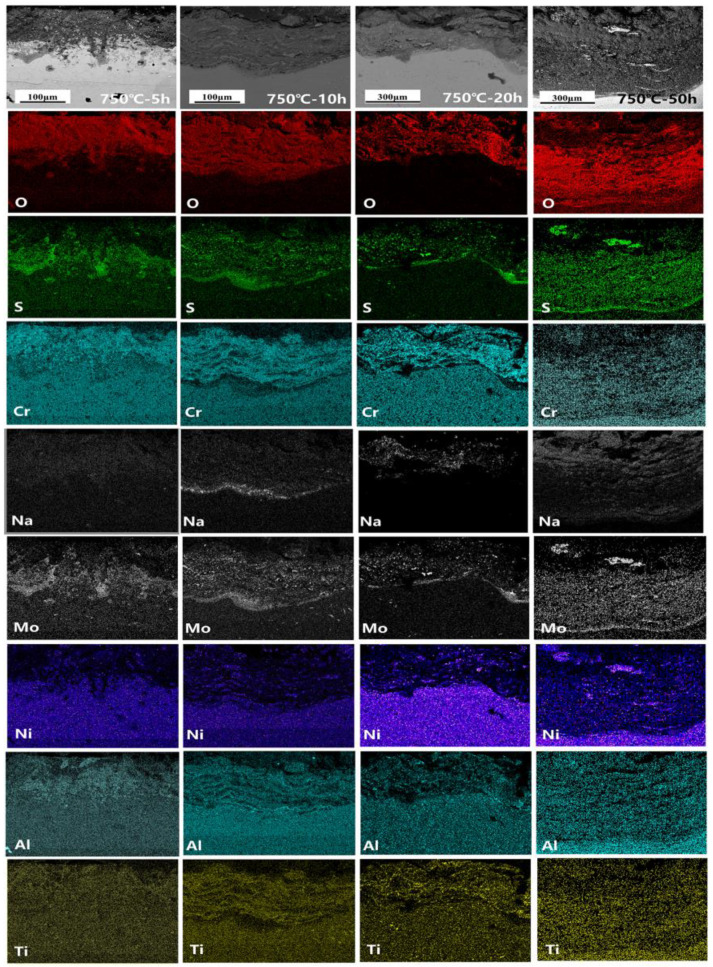
Cross-sectional morphology and elemental distribution of alloy at 750 °C.

**Figure 6:**
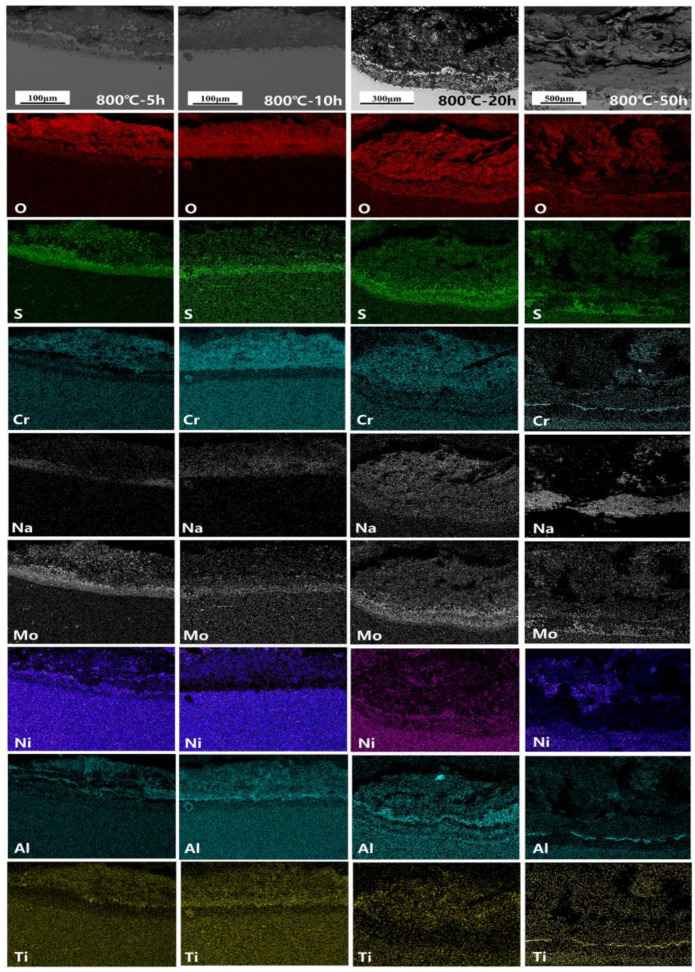
Cross-sectional morphology and elemental distribution of alloy at 800 °C.

The cross-section morphology and element distribution images of the specimens under corrosion are shown in Figure 4, Figure 5 and Figure 6. The oxide scale, sulfide scale, and matrix organization are the three separate sections that make up the cross-section. In combination with the XRD results, this oxide scale is primarily in the form of Cr_2_O_3_ and Al_2_O_3_, whose presence can be used as a protective oxide to slow down the oxidation rate [28,29]. The Ni element also progressively diffuses to the surface, generating nickel (II) oxide (NiO) and Ni_3_S_2_ inclusions in the Cr_2_O_3_ oxide scale as the reaction time increases. In the inner scale of the corrosion product, the S and Mo elements are primarily mixed to create Molybdenum disulfide (MoS_2_). As is shown in Figure 5 and Figure 6, the Al elements exhibit a more positive response. This means that, at a higher temperature, the protection of the outer oxidation product is worse. The damage caused by the corrosion time extension to 50 h is severe: the thickness of the alloy matrix decreases significantly due to the gradual diffusion of the alloying elements Ni, Cr, Al, and Ti to form a thicker oxide scale. The Mo elements are found in the inner surface scale and throughout the damage scale.

**Figure 7:**
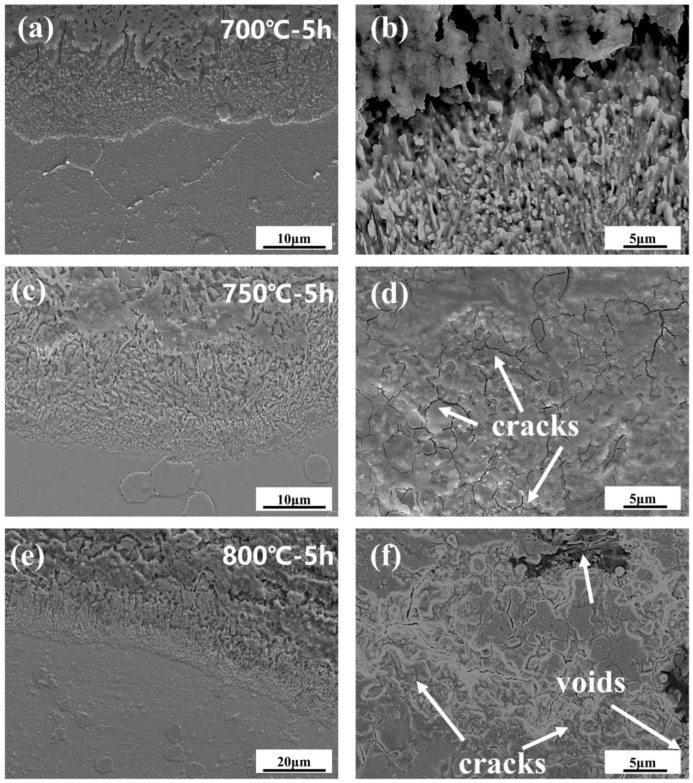
Cross-sectional morphology of the oxidized sulfide matrix at the early stage of corrosion at 700–800 °C. (**a**,**b**) 700 °C-5 h, (**c**,**d**) 750 °C-5 h, (**e**,**f**) 800 °C-5 h.

The cross-sectional morphology of the early stage of corrosion is shown in Figure 7. The cross-section morphologies of the damage scales are affected differently by different temperatures. For example, the oxide and sulfide scales exhibit a clear delamination structure but no visible cracks or voids at 700 °C-5 h. At 750 °C-5 h, there are visible crack distributions in the oxide scales. Cracks and a few voids appear in the cross-section morphologies of damage scales at 800 °C-5 h. As the temperature increases, the sulfide scale becomes gradually thickened. This allows the metal to react with low-melting-point sulfides such as Ni_3_S_2_. It has a lower melting point and more crystal flaws with co-crystals, which enables the elements to diffuse quickly and creates a weaker scale that is more prone to damage [30,31]. Therefore, when the initial temperature increases, the corrosion of the oxide scale density gradually decreases until 800 °C when there are cracks and voids.

### 3.5. Comparison of Changes in Tensile Properties Before and After Hot Corrosion

**Figure 8:**
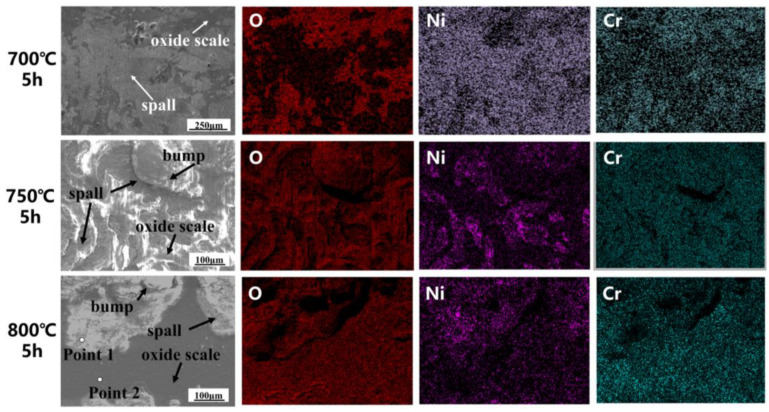
Analysis of the main components of the corroded surfaces at 700, 750, and 800 °C-5 h.

The morphological observation and composition analysis of the corrosion products on the surface is shown in Figure 8. At 700 °C, the surface comprises a spalling defect, an oxide scale, and minor bumps. As the temperature rises, the bumps progressively get bigger, unite at 750 °C, and show many cracks at 800 °C. Because of oxidation and matrix element depletion, cracks may originate from the oxidized surface and grow quickly at high temperatures in service [32], hence decreasing the overall strength and causing premature failure. Figure 8 reveals some spalling of the oxide scale and that the Cr and Oxygen (O) elements predominate in the oxide protective scale. Bump defects are mainly composed of Ni and Cr oxides, and as the temperature increases here, the Cr contrast is relatively reduced. As shown in Table 2, the Ni element ratio is significantly higher in bump defects than in surface oxide scale structure. Because the “oxidation-sulfidation” mode of the hot corrosion reaction allows corrosive elements to pass through the oxidation scale to alloy, as a result, the Cr_2_O_3_-based oxide scale is inevitably be destroyed, resulting in a small number of defects in the composite oxide scale.

**Figure 9:**
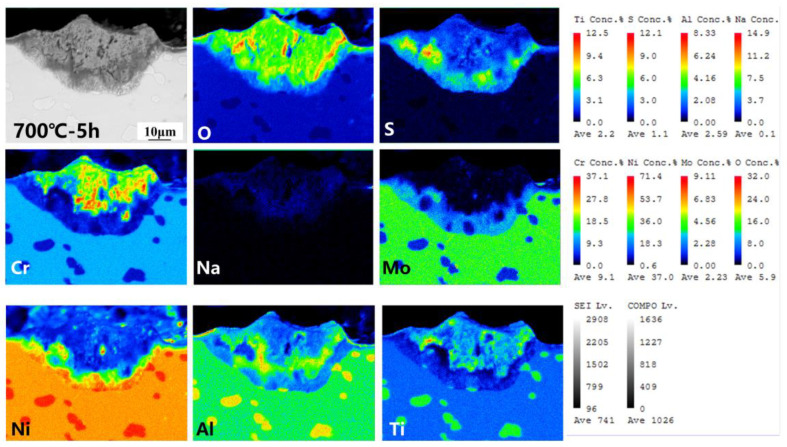
Element distribution of experimental alloy after hot corrosion at 700 °C-5 h.

**Figure 10:**
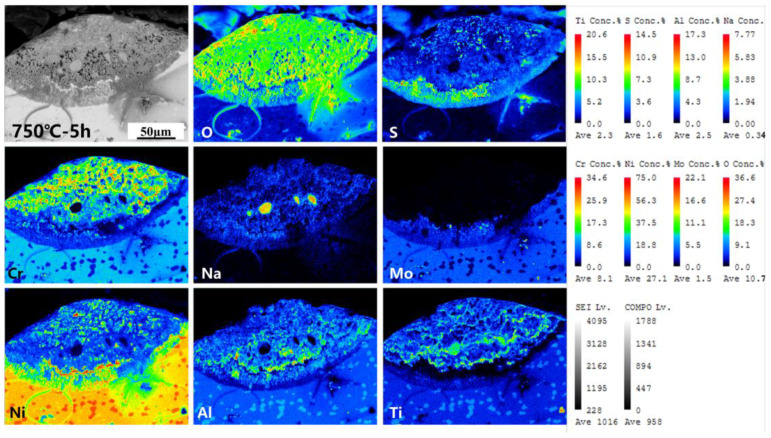
Element distribution of experimental alloy after hot corrosion at 750 °C-5 h.

**Figure 11:**
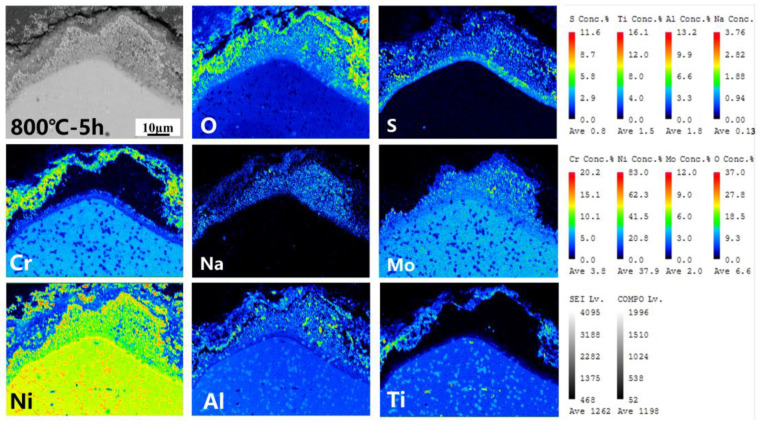
Element distribution of experimental alloy after hot corrosion at 800 °C-5 h.

The distribution of element components in the damaged scale is illustrated in Figure 9, Figure 10 and Figure 11. A more detailed view of a small amount of the Al_2_O_3_ subscale between Cr_2_O_3_ and MoS_2_ at 700 °C and 750 °C is also visible. Unlike in the studies by Yu et al. [33] and Han et al. [34], the alloy does not have a significant depleted scale of Cr present in the matrix organization after hot corrosion; in the present experiments, it is mainly replaced by MoS_2_. The Ni elements are also visible in the aggregation of the inner surface scale and in a small portion of the diffusion phenomenon to the surface. Notably, the Ni elements exhibit a significant outward diffusion at 800 °C-5 h, increasing the percentage of Ni sulfides and oxides in the damage scale. In contrast, the Ti elements show a positive oxidative response by progressively diffusing into the outer surface scale. This indicates that the Cr_2_O_3_-based oxide scale provides some protection during the early stages of hot corrosion, but the protective effect is gradually reduced with temperature with extensions.

**Figure 12:**
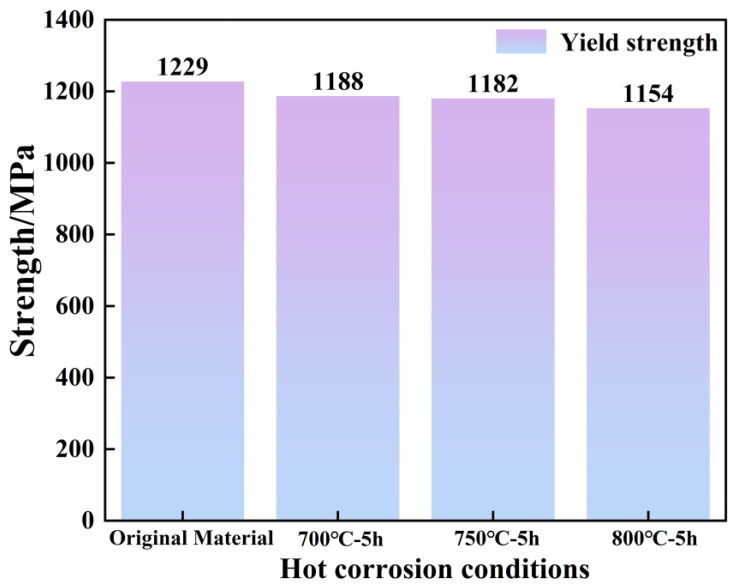
Trends in room-temperature yield strength properties under various conditions.

As can be seen in Figure 12, the yield strengths exhibit a declining trend during hot corrosion. The tensile yield strength of the alloy decreases by 3.3, 3.8, and 6.1% at 700, 750, and 800 °C, respectively, from the standard heat-treated state specimens. A total of 800 °C has a greater effect on the strength properties of the alloy. This trend is mainly associated with microstructures and the element consumption of specimens. This indicates that the extent of sulfide damage to the protective layer increases as the thermal corrosion temperature increases, which is consistent with the EPMA results above. Although just three temperature point specimens were used to assess the mechanical properties of the corroded alloys, the trend of how the damaged structure affected the strength characteristics was identifiable.

## 4. Discussion

### 4.1. Corrosion–Oxidation Mechanism

**Figure 13:**
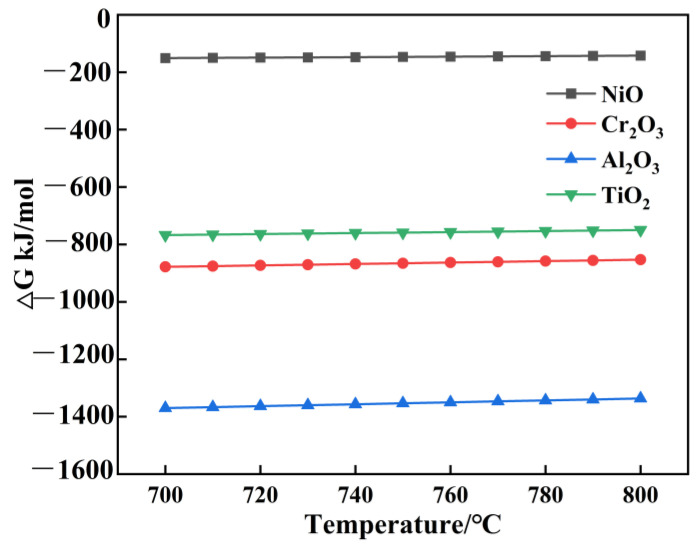
Total of 600–800 °C Oxide Generation Gibbs Free Energy Change.

The corrosion–oxidation mechanism can be summed up based on the discussion above. The oxide generation free energy change calculated by HSC Sim in 600–800 °C is illustrated in Figure 13. It has been demonstrated that the element Cr of binary alloys (such as Ni-Cr) significantly affects the oxidation rate constants of alloys. The oxidation rate constants Kp for Al_2_O_3_ and Cr_2_O_3_ are several orders lower than for NiO and CoO. As a result, it is easier to form a protective Al_2_O_3_ or Cr_2_O_3_ scale on the alloy surface [35]. The most active elements react with oxygen to form oxides that grow inward from the surface of the alloy, which is called internal oxidation [36]. The Al element shows the most significant driving force due to the lowest formation energy, which preferentially promotes the growth trend of Al_2_O_3_. However, the diffusion rate of Cr is much higher than that of Al; Cr_2_O_3_ grows at a higher rate than the Al_2_O_3_ oxide scale, so the early stage of oxidation preferentially forms Cr_2_O_3_, and Al_2_O_3_ exists in the form of internal oxidation. At the same time, the Cr element in superalloys primarily resides as a Cr_2_O_3_ outer surface oxide because it is more active than NiO, causing the replacement reaction [37].

3NiO + 2Cr → 3Ni + Cr_2_O_3_ (3)

With the deepening corrosion conditions, the Cr_2_O_3_ scale becomes loose and even cracks; at this time, the Ni element as a matrix element quickly diffuses to the surface, furthering the bump enrichment and gradual thickening. Secondly, a tiny amount of TiO_2_ and Al_2_O_3_ is formed on the surface as the Ti and Al elements progressively diffuse outward. Furthermore, the produced spinel-structured oxides, such as nickel (II) chromite (NiCr_2_O_4_), might further deteriorate the surface densification and increase the degree of corrosion [15].

NiO + Cr_2_O_3_ → NiCr_2_O_4_ (4)

In addition to the oxidation mechanism, however, the role of the corrosive medium should not be ignored. According to pertinent research, while corrosion occurs in an environment with 75% Na_2_SO_4_ + 25% NaCl molten salt, the molten NaCl dissolves the protective Cr_2_O_3_ scale by penetrating the oxide scale and reacting directly with Cr_2_O_3_. On the other hand, the competition of dissolution and forming finally results in a loose oxide scale when the molten salt combines with oxides like Cr_2_O_3_ [38,39,40]. The reaction equations are as follows:

4NaCl + O_2_ → 2Cl_2_ + 2Na_2_O (5)

Na_2_SO_4_ → SO_3_ + Na_2_O (6)

Na_2_SO_4_ → Na_2_O_2_ + SO_2_ (7)

Cr_2_O_3_ + 3Na_2_O_2_ → 2Na_2_CrO_4_ + Na_2_O (8)

2Cr_2_O_3_ + 3O_2_ + 4Na_2_O → 4Na_2_CrO_4_ (9)

4NaCl + Cr_2_O_3_ + 5/2O_2_ → 2Na_2_CrO_4_ + 2Cl_2_ (10)

**Figure 14:**
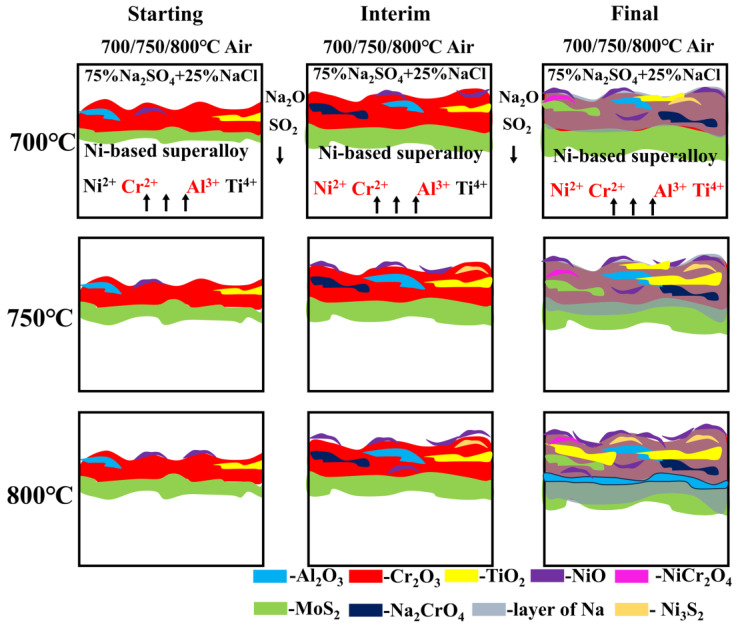
Corrosion–oxidation process schematic diagram.

So, the primary factor in the deepening of corrosion–oxidation damage in high temperatures is the lapse of protective oxides, whereby the migration of Ni and Cr elements to the surface is the primary cause of the oxide scale thickening. The oxidation response of the Al and Ti elements is observed to be more positive at higher temperatures and longer corrosion times. This phenomenon is also one of the factors responsible for the thickening of the corrosion oxidation product scale. Additionally, the depletion of alloy elements, such as Ti and Al, could promote crack initiation, ultimately leading to the mechanical property degradation of the alloy. In summary, the corrosion and oxidation mechanism diagram is shown in Figure 14.

The percentage of the sulfide scale exhibits a growing trend with increasing temperature (700 °C-50 h: 30 μm, 750 °C-50 h: 33 μm, and 800 °C-50 h: 208 μm). The formation of the sub-surface scale of the more dense Al_2_O_3_ also has a specific reduction in the diffusion of matrix components to the exterior at the hot corrosion temperature of 800 °C. However, the outer Cr_2_O_3_ oxide scale repeatedly undergoes a “dissolution-regeneration” process, which results in the penetration of the S elements into the oxide scale and the matrix. This leads to the deeper penetration of sulfide damage, and a complicated oxidation–sulfidation damage structure is produced. The following is the reaction equation: sulfur damage predominates in this molten salt environment at temperatures of 800 °C, whereas oxidation damage predominates at temperatures of 700 °C and 750 °C, with sulfide damage as a supplement.

3Ni + 2S → Ni_3_S_2_ (11)

4Na_2_SO_4_ + 18Ni + 2Cr_2_O_3_ + 3O_2_ → 4Na_2_CrO_4_ + 12NiO + 2Ni_3_S_2_ (12)

### 4.2. Formation of Crack and Spalling

Spalling can potentially come from oxide internal stresses. One type of growth stress is caused by the growth and formation of the oxide. The other is thermal stress generated from different coefficients of thermal expansion (CTEs) in the metal/oxide system [15,41]. The volume difference of oxides and metal is the primary source of growth stresses. The volume difference at the scale/alloy contact where the oxide forms can be represented by the Pilling–Bedworth ratio (PBR):

(13) $$PBR=\frac{v_{oxides}}{v_{alloy}}$$

(14) $$\varepsilon =\sqrt[{3}]{PBR}-1$$

(15) $$\sigma_{G}=\varepsilon Eoxides$$

Table 3 displays the relevant parameters and calculations. *V_oxides_* and *V_alloy_* are the oxide and metal volumes, respectively, and *E_oxides_*, *ε*, and *σ_G_* stand for the oxide Young’s modulus, growth strain, and stress.

In the cooling process, thermal stresses arise. The different thermal expansions in the oxide scale and the alloy cause thermal stresses. Consequently, the following formula can be used to determine the thermal stress or *σ_T_*:

(16) $$\sigma_{T}=Eoxides(\alpha_{alloy}-\alpha_{oxides})∆T$$

where ∆*T* is the difference in temperature during cooling, and *α_alloy_* and *α_oxides_* are the respective coefficients of the thermal expansion of oxides and metal. The computed temperatures were set to 700, 750, and 800 °C. The coefficients of thermal expansion of the alloys were determined by the software JmatPro7.0; *α_alloy_* was found to be 1.52 × 10^−6^ K^−1^. Table 4 displays the parameters and the results of the computation.

From computations, thermal stresses are orders of magnitude smaller than the growth stresses. Research has revealed that [43,44] when PBR > 1, stress can be called compressive stress in the oxide scale. On this basis, it can be concluded that the cracking and spalling of the oxide are caused by growth stresses and promoted by thermal stresses, which explains how oxides crack and spall.

**Figure 15:**
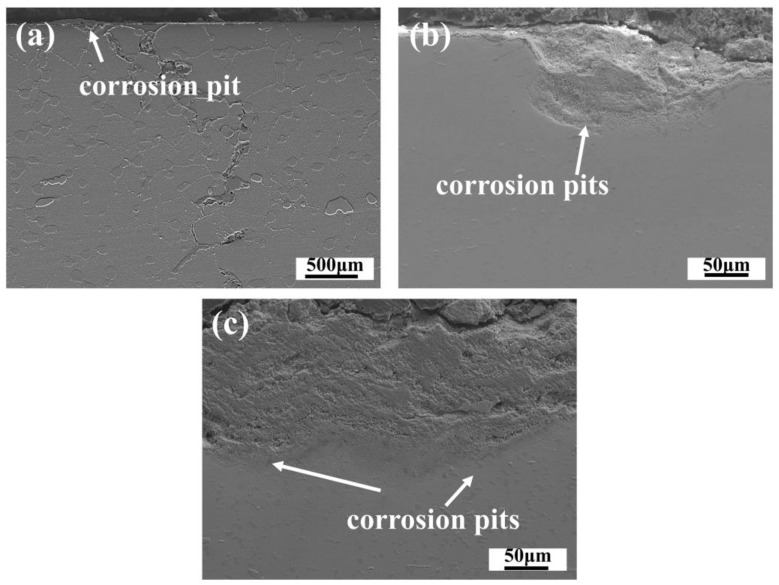
Corrosion pit morphology. (**a**) 700 °C-5 h, (**b**) 750 °C-5 h, (**c**) 800 °C-5 h.

In this study, the experimental alloy shows rapid corrosion rate constants because of its relatively low Cr and Al element content. The oxidation of the Cr and Ni elements accounts for a significant proportion. During the hot corrosion phase, the Ni element diffusion primarily occurs when the protective Cr_2_O_3_ is generated and dissolved. This leads to the O element invasion process, which quickly produces NiO and thickens the oxide scale quickly. Simultaneously, the S element intrusion reacts with the Mo and Ni elements in the matrix to form an inner scale MoS_2_ sulfide scale and Ni_3_S_2_ inclusions. The alloy matrix corrodes rapidly due to the outward diffusion of Ni, Cr, and other elements, forming corrosion pits on the matrix surface. As temperatures approach 700–800 °C, contaminants such as salt from the intake air, engine components, and combustion may erode the protective oxides on these discs’ surface. When the salt locally reduces these oxides, pits appear, and the protection is lost [45]. Unlike usual investigations, the larger thickness of the corrosion–oxidation scale prevented the appearance of pits on the surface. The research of Timothy [46] is similar to our findings, revealing that the oxide scale was occasionally kept in and on the pits. Figure 15 illustrates the pits in the early phases of corrosion at 700, 750, and 800 °C, covered by an oxide scale. With the temperature increase, the pit deepened. Research demonstrates that [47] at the bottom of the corrosion pit surface, localized corrosion is formed and grows along the grain boundaries. The sensitive grain boundaries cause very early fatigue crack initiation at 704 °C. Eventually, the pit becomes severely damaged due to the matrix microstructure.

### 4.3. Mechanism of Competition for the Element Diffusion

Currently, conventional heat-resistant alloys obtain oxidation resistance by the diffusion of Cr outward to form a dense Cr_2_O_3_ scale on the surface. But when exposed to high temperatures and pressures in a water vapor environment, Cr_2_O_3_ readily forms the unstable hydroxide chromium (IV) hydroxide (CrO_2_ (OH)_2_), which is unstable and volatilized. This severely compromises the integrity of Cr_2_O_3_ and eliminates the protective function of the oxide scale. Many researchers focus on the Al_2_O_3_-type oxide scale, which has the same corundum structure, density, and strong bonding force as the matrix to tackle volatility at high temperatures. The Al_2_O_3_ type has a lower oxidation rate than that of the Cr_2_O_3_ type. This is primarily due to the slower growth rate, higher thermodynamic stability, and higher antioxidant capacity in the high-temperature water vapor environments of Al_2_O_3_. Additionally, in many service environments, the effect of oxidation protection is more prominent [48]. Ni-Cr-Al alloys can be classified into three categories based on their oxidation behavior: a non-protective external NiO scale with discontinuous Cr_2_O_3_ and Al_2_O_3_ intrusions is called a type I alloy; type II alloys combine a discontinuous Al_2_O_3_ intrusion phase suboxide scale with an external Cr_2_O_3_ continuous oxide scale; and type III alloys create an uninterrupted Al_2_O_3_ exterior oxide scale [49]. Combining the experiment results in this study, it is clear that the experimental alloy does not exhibit a precise continuous Al_2_O_3_ scale at 700–750 °C. The increase in temperature leads to the gradual appearance of the continuous internal oxide scale structure of Al_2_O_3_ at 800 °C. In a gas turbine engine, the current temperature of the turbine disk typically does not exceed 750 °C. If polycrystalline superalloys can form Al_2_O_3_ (type III) instead of Cr_2_O_3_, experimental alloys could benefit more fuel-efficient gas turbine engines in the future. Consequently, the computational approach [50] has been usually used to predict the tendency toward preferentially formed protective oxides. Wagner [51] assumed that the transition from internal to external oxide formation occurs if the solute concentration is high enough to cause the volume fraction of internal oxides to be more significant than a critical volume fraction. However, with regard to Al_2_O_3_, an external protective oxide scale appears to fail in the experiment. This requires the help of the formula for the lowest solute concentration needed in a binary A–B system to initiate external oxidation:

(17) $$N_{B}^{O}={\left[f\left(\frac{V_{m}}{V_{0}x}\right)\pi \frac{N_{O}^{S}}{2\nu }\frac{D_{O}}{D_{B}}\right]}^{\frac{1}{2}}$$

(18) $$N_{0}^{s}=8.3\cdot \exp\left[\frac{-55\frac{kJ}{mol}}{RT}\right]$$

(19) $$D_{O}=1.7\times 10^{-5}\cdot \exp\left[\frac{-90\frac{kJ}{mol}}{RT}\right]$$

(20) $$D_{B}=1.1\cdot \exp\left[\frac{-249\frac{kJ}{mol}}{RT}\right]$$

where $N_{B}^{O}$ represents the minimum critical concentration of solute *B* required for external oxidation, and *V_m_* denotes the molar volume of the alloy at a specific temperature (cm^3^·mol^−1^). This portion is determined to be 7.20 (cm^3^·mol^−1^) at 800 °C calculated by Jmatpro 7.0. The molar volume of Al_2_O_3_ (*V_ox_*), which stands for the molar volume of oxides (cm^3^·mol^−1^), is computed using the standard density and molecular weight values from Refs. [26,52], and its value is 25.575 cm^3^·mol^−1^. $N_{O}^{S}$ is the solubility of oxygen in the alloy, expressed as a mole fraction; Equation (18) illustrates the O element in the Ni-based component of the solubility; *D_O_* denotes the diffusion rate of Ni in the alloy, and it can be roughly calculated using the diffusion rate of O in the Ni matrix, as indicated by Equation (19) [53]. According to Equation (20), *D_B_* is the solute diffusion rate of Al in the Ni matrix (in cm^2^·s^−1^) [54]. The value of *v* can be defined as the ratio of element O stoichiometry to element *B*, which is, for example, 1.5 in the case of Al_2_O_3_. It is pretty realistic to use 0.3 as the volume fraction f of the internal oxides based on the research of Nesperson [55] and Pettit [56]. Table 5 displays the computation and simulation results.

Based on theoretical calculations, the Al_2_O_3_ oxide scale is generated as an external oxide scale when the alloy system contains 9.78% (wt %) of Al. Consequently, under 800 °C hot corrosion conditions, the continuous Al_2_O_3_ oxide scale forms only in the inner scale and thickens gradually over time. However, it is worth noting that oxygen filling decreases under hot corrosion conditions in molten salts, and the Al external concentration may increase.

### 4.4. Corrosive Properties Damage

**Figure 16:**
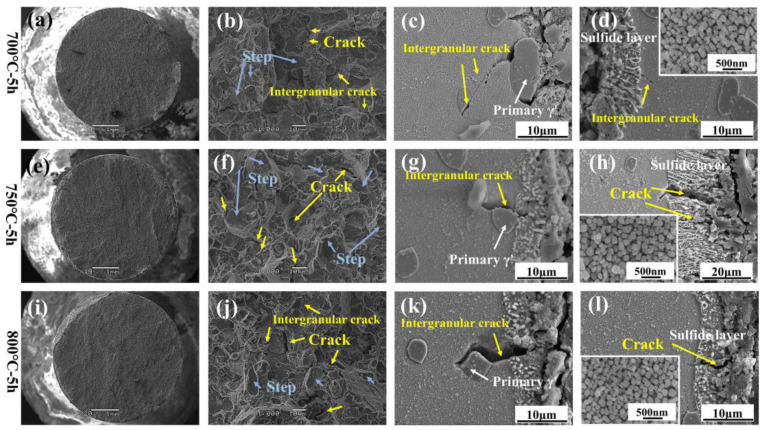
Fracture morphology of RT tensile specimens under different corrosion conditions. (**a**,**b**,**e**,**f**,**i**,**j**) fracture morphology, (**c**,**g**,**k**) cracks between primary γ′ and matrix, (**d**,**h**,**l**) cracks in sulfide scale.

The fracture morphology of RT tensile specimens under different corrosion conditions is shown in Figure 16. The fracture morphology is flat and microscopically characterized by intergranular brittle fractures in the presence of cleavage steps. These features include blocky steps, river patterns, and intergranular cracks. The elongation exhibits a declining trend when the cracks proliferate during the tensile process, even when the strength properties are not severely affected [15]. As shown in Figure 16c,d,g,h,k,l, the longitudinal section near the tensile fracture showed significant sulfide scale cracking. This cracking source extended to the matrix and affected the mechanical characteristics by causing intergranular cracks. Furthermore, more severe cracks in the matrix occur due to the rapid expansion along the phase boundary between the primary γ′ and the matrix.

**Figure 17:**
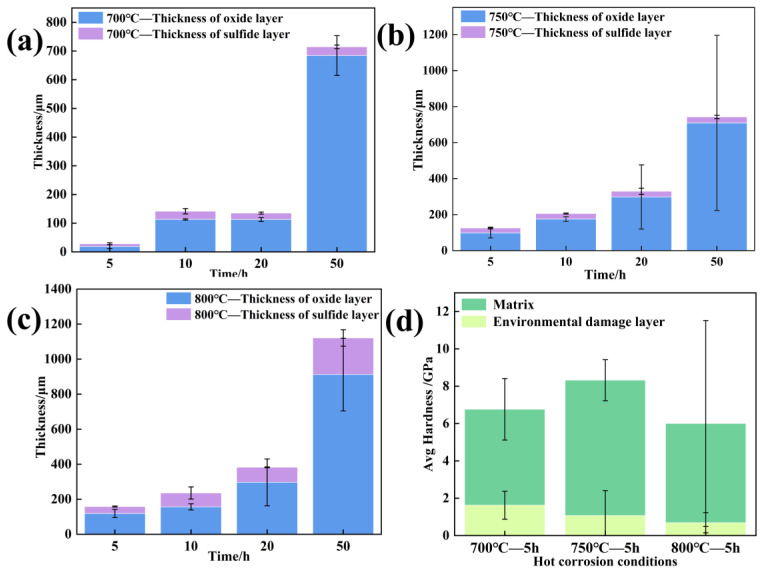
Characteristics of environmental damage scales and matrix under different hot corrosion conditions. (**a**–**c**) Thickness and (**d**) hardness.

The hardness of the substrate and oxide–corrosion scale under various conditions were determined by the nanoindentation test method in Figure 17d. If the indentation depth is set at 100 nm, the hardness of the sulfide-damaged scale is significantly lower than that of matrix. This suggests that there will be more plastic deformation in this area during the deformation process, leading to significant strain localization, which is the reason that causes the sulfide scale to crack. According to Figure 17a–c, the loss of properties is marginally less at 700 °C due to the much thinner damage scale than at 750 and 800 °C under 5 h conditions. Due to the thin thickness of the corrosion early damage scale at 700 and 750 °C, the thickness of the sulfide scale does not differ much, so the yield strength decline is slightly lower and more similar. When the thickness of the sulfide scale is more significant at 800 °C, the strength properties are of a more considerable degree of attenuation.

The PBR value of the oxide scale is greater than one, so the compressive stress placed on the matrix theoretically can protect the material and increases the role of the force area. However, in extremely hot corrosion conditions, the oxide scale is substantially damaged, and the beneficial effects of the scale are not realized [57]. In addition, there are localized pits on the alloy surface because of inhomogeneous hot corrosion [58]. The force area then shrinks to create a localized stress concentration, which has an additional impact on the mechanical characteristics [46,47].

In the foundation of the corrosion–oxidation mechanism, the following impacts of the mechanical characteristics of the hot corrosion process are summarized: (1) since the spalling of the surface damage scale may accelerate the substrate corrosion, the stress concentration increases, and the effective bearing area of the specimen is reduced; (2) surface pits and bumps are created by the cracking and spalling of the hot corrosion scale, which concentrates stress and speeds up the formation of cracks; (3) under load, microcracks propagate into the matrix through the sulfide scale, ultimately leading to fracture and significantly influencing properties; (4) strain localization and accelerated crack initiation may result from plastic deformation which is exacerbated by the decreased hardness of the sulfide scale.

## 5. Conclusions

This study systematically investigates the hot corrosion behavior of a new nickel-based high-temperature alloy under different conditions and its effect on the alloy properties. Analyzing the composition of corrosion products, the damage mechanisms, and the impact of corrosion on properties provides crucial theoretical support for applying the alloy in high-temperature environments. However, thermal corrosion is a complex multifactorial process, and future research can further improve the corrosion resistance of alloys by developing new protective coatings, optimizing alloy compositions, and simulating corrosive environments that are closer to actual working conditions. These research directions will not only help solve the current corrosion problems faced but also provide broader prospects for designing and applying superalloys. The main conclusions of this paper are as follows:

1. The damage scale is composed of inner sulfides and external oxides, and the hot corrosion element diffusion mechanism is governed by the diffusion of the Cr, Ni, Al, Ti, and Mo elements. At 700 °C and 750 °C, oxidized damage predominates, and at 800 °C, sulfurized damage takes center stage.
2. Since the crucial generation conditions of external oxides are not met, the Al element content appears as a sub-surface scale at 800 °C, with Cr_2_O_3_ making up the majority of the surface scale oxides.
3. During steady-state hot corrosion, the sharp increases in growth stress caused by the formation of oxides and spinels are the leading cause of the cracking and spalling of the composite oxide scale. With the increase in corrosion, there also exists a damage mechanism of dissolution–regeneration–dissolution in the surface oxide organization.
4. The main effects of hot corrosion on the alloy’s tensile properties are the exfoliation cracking of the surface oxide scale, the corrosion pit on the matrix, and the sulfide scale as a source of fractures.

## Figures and Tables

**Table 1:** Main chemical composition in experimental alloy (wt %).

C	Cr	W	Mo	Ti	Nb	Al	Co	V	Ni
0.05	11.3	3.0	4.5	2.6	3.1	3.8	14.7	0.6	Balanced

**Table 2:** EDS compositional analysis elemental content.

	O	Al	S	Ti	Cr	Ni
Point 1	37.69	1.81	0.49	2.55	15.22	42.24
Point 2	48.98	1.93	2.91	7.64	32.37	6.76

**Table 3:** PBR, *E_oxide_*, and *σ_G_* of oxides in oxide scale [13].

Oxides	Cr_2_O_3_	NiCr_2_O_4_
*PBR*	2.07	2.05
*E_oxides_*	260	233
*σ_G_*	71.35 GPa	62.99/GPa

**Table 4:** α_alloy_ and α_oxide_ of oxides and alloy [13,42].

Oxides	Cr_2_O_3_	NiCr_2_O_4_
$\alpha_{oxides}$	9.6 × 10^−6^ K^−1^	7.6 × 10^−6^ K^−1^
$\alpha_{alloy}$	15.2 × 10^−6^ K^−1^	15.2 × 10^−6^ K^−1^
$\sigma_{T}$ (700 °C)	0.99 GPa	1.20 GPa
$\sigma_{T}$ (750 °C)	1.06 GPa	1.29 GPa
$\sigma_{T}$ (800 °C)	1.13 GPa	1.38 GPa

**Table 5:** Parameter values for the lowest critical Al content needed to cause the alloy to externally oxidize at 800 °C.

*V_m_*	$N_{0}^{s}$	*D_O_*	*D_B_*	$N_{B}^{O}$
7.20	1.75 × 10^−4^	7.07 × 10^−10^	8.34 × 10^−10^	9.78%

## Data Availability

The original contributions presented in this study are included in this article. Further inquiries can be directed to the corresponding author.
